# Spatial Models of Abundance and Habitat Preferences of Commerson’s and Peale’s Dolphin in Southern Patagonian Waters

**DOI:** 10.1371/journal.pone.0163441

**Published:** 2016-10-26

**Authors:** Natalia A. Dellabianca, Graham J. Pierce, Andrea Raya Rey, Gabriela Scioscia, David L. Miller, Mónica A. Torres, M. Natalia Paso Viola, R. Natalie P. Goodall, Adrián C. M. Schiavini

**Affiliations:** 1 Centro Austral de Investigaciones Científicas (CADIC-CONICET), Ushuaia, Tierra del Fuego, Argentina; 2 Museo Acatushún de Aves y Mamíferos Marinos Australes, Ushuaia, Tierra del Fuego, Argentina; 3 Oceanlab, University of Aberdeen, Newburgh, Aberdeenshire, Scotland, United Kingdom; 4 CESAM & Departamento de Biologia, Universidade de Aveiro, Aveiro, Portugal; 5 Integrated Statistics, Woods Hole, Massachusetts, United States of America; 6 Centre for Research into Ecological and Environmental Modelling, and School of Mathematics and Statistics, University of St Andrews, St Andrews, Scotland, United Kingdom; University of Missouri Columbia, UNITED STATES

## Abstract

Commerson’s dolphins (*Cephalorhynchus c*. *commersonii*) and Peale’s dolphins (*Lagenorhynchus australis*) are two of the most common species of cetaceans in the coastal waters of southwest South Atlantic Ocean. Both species are listed as Data Deficient by the IUCN, mainly due to the lack of information about population sizes and trends. The goal of this study was to build spatially explicit models for the abundance of both species in relation to environmental variables using data collected during eight scientific cruises along the Patagonian shelf. Spatial models were constructed using generalized additive models. In total, 88 schools (212 individuals) of Commerson’s dolphin and 134 schools (465 individuals) of Peale’s dolphin were recorded in 8,535 km surveyed. Commerson’s dolphin was found less than 60 km from shore; whereas Peale’s dolphins occurred over a wider range of distances from the coast, the number of animals sighted usually being larger near or far from the coast. Fitted models indicate overall abundances of approximately 22,000 Commerson’s dolphins and 20,000 Peale’s dolphins in the total area studied. This work provides the first large-scale abundance estimate for Peale’s dolphin in the Atlantic Ocean and an update of population size for Commerson’s dolphin. Additionally, our results contribute to baseline data on suitable habitat conditions for both species in southern Patagonia, which is essential for the implementation of adequate conservation measures.

## Introduction

Commerson’s dolphin *Cephalorhynchus c*. *commersonii* and Peale’s dolphin *Lagenorhynchus australis* are two of the most common small cetaceans inhabiting coastal waters of the southwest South Atlantic Ocean [1–3]. With a geographic distribution restricted to southern South America, both taxa co-occur along the Patagonian shelf, mainly from Golfo San Jorge (44°S) to Tierra del Fuego (56°). Commerson’s dolphin has been reported along the Atlantic coasts from about 41°30′S° to near Cape Horn (56°S), as well as from the central and eastern Strait of Magellan and the Malvinas (Falkland) Islands [1, 4]. It inhabits shallow waters of the continental shelf (where tidal amplitudes are large) as well as areas near and within the mouths of rivers, bays and estuaries [1]. Peale’s dolphin is found along both coasts of South America, from 33°S on the Pacific [5] and from 38°S, including the Malvinas (Falkland) Islands and the Namuncurá (Burdwood) Bank, on the Atlantic to south of Cape Horn (59°S). However, sightings are more numerous from Valdivia in Chile (38°S) and within the waters of the Golfo San Jorge in Argentina to the south of Tierra del Fuego [2, 6, 7]. It occurs in two different habitats within its distributional range: 1) protected channels and fjords in southern Chile and 2) shallow continental shelves in the northern portion of its distribution in Chile and throughout most its range in Argentina [6, 8].

Similarly to other coastal small cetaceans, these species face various threats, many of them related to anthropogenic activities. Commerson’s dolphin is the small cetacean with highest level of incidental mortality in artisanal coastal gill nets in Santa Cruz and Tierra del Fuego provinces, Argentina [4, 9–11]. In central and northern Patagonia of Argentina, interactions with mid-water trawl fisheries have also been reported [12]. It is one of the target species of dolphin watching activities in northern Patagonia [13]. Peale’s dolphin is also affected by by-catch in artisanal fisheries in Santa Cruz and Tierra del Fuego provinces, although in a lower proportion than Commerson’s [4, 10, 14].

In addition, both Commerson’s and Peale’s dolphin could be influenced by climate change. MacLeod [15] postulated that the distributional range of dolphins that inhabit shallow and cold waters would be reduced by an increase of sea surface temperature. The potential impact of these threats cannot be assessed without adequate information on population sizes and trends. This lack of information is one of the main reasons that both species are listed as “Data Deficient” by the IUCN [16, 17]. Commerson’s dolphin abundance has been estimated (mostly at small scales) using both distance sampling [18–21] and mark-recapture methods [22, 23]. To date, only one large-scale population estimate of this species is available, from the early 2000s [24].

For Peale’s dolphin, there is an estimation of around 2400 individuals for the Magellan region and some 200 animals in a local population off Chiloe [25, 26] but no abundance estimates are available for the species in the Atlantic Ocean. Additionally, studies on spatial distributions and habitat preferences of Commerson’s and Peale’s dolphins are also scarce and generally limited to small parts of their range. For Commerson’s dolphin, studies were conducted mainly in northern and central Patagonia of Argentina [3] while for Peale’s dolphin all previous studies have been carried out along the Chilean coast [7, 26, 27].

The geographic distributional range of marine mammals is generally thought to be determined by water temperature whereas distribution at small scales is often associated with oceanographic conditions which affect the distribution of their prey [14, 28, 29]. Thus, habitat selection by top predator species in marine ecosystems is generally defined by physical, chemical and biological variables, generating a differential use of areas within the range of their distributions [28, 30]. Detailed information on the areas preferred by a species, at different spatial scales, is essential for understanding ecology and life history features, and crucial for conservation [31]. Any natural or anthropogenic environmental changes within preferred areas could have profound impacts on the distribution and abundance of these species, causing different responses in each [32].

Spatially explicit models enhance our knowledge of relationships between biological population densities and the environment, allowing us to investigate which factors affect distribution and abundance. Biogeographic variables such as sea surface temperature, depth, chlorophyll *a* and thermal fronts are frequently used as proxies of prey distribution and have been related to distribution of marine mammal in numerous studies ([3, 29, 33–35], among others).

Here we used generalized additive models (GAMs) to build spatially explicit models using observed counts as a response variable in a manner similar to Density Surface Models (DSMs) [36, 37]. Building an explicit spatial model means that we do not require a randomized survey design (though of course, this would have been beneficial), allowing the use of data recorded from platforms of opportunity [37]. This is particularly useful given the cost and logistic complexities of studying cetaceans at sea.

The main goal of this study was modelling the abundance for Commerson’s and Peale’s dolphins in sub-Antarctic waters of the southwest South Atlantic Ocean in relation to environmental variables. Results of this study give a better understanding of the habitat conditions required for both taxa within their distributional range along the Patagonian shelf, especially in southernmost Patagonia.

## Materials and Methods

### Study area and data collection

**Fig 1:**
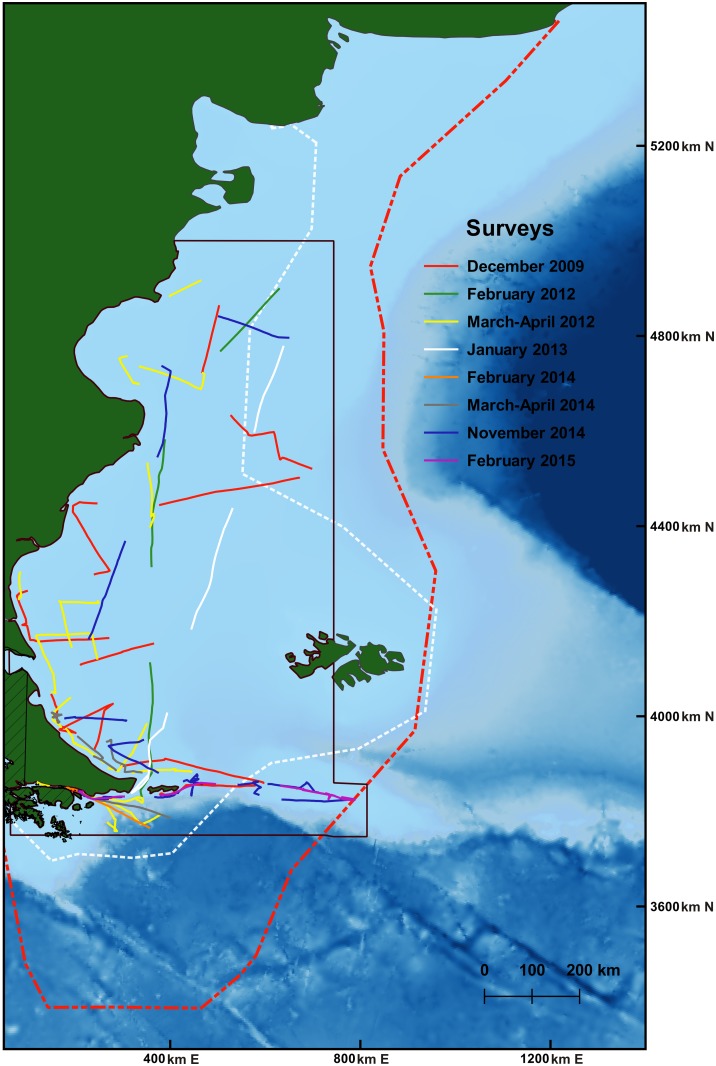
Tracks of the different scientific cruises surveys during austral summer and fall months. Each single line of each cruise represents one day of survey effort. Black polygon indicates the region of study analyzed. White polygon indicates the primary range of distribution of Commerson’s dolphin in the southwest South Atlantic Ocean. Red polygon indicates the primary range of distribution of Peale’s dolphin in the southwest South Atlantic Ocean.

Abundance data on Commerson’s and Peale’s dolphin were collected during eight scientific cruises on board of the vessels *R/V Puerto Deseado* and *Tango SB-15* during austral summer and fall months (November-April) between 2009 and 2015, along the Patagonian shelf (Fig 1). Research was authorized by the Argentine Federal Government through the National Scientific and Technical Research Council (CONICET).

Cetacean sightings were recorded on a portable handheld computer with integrated GPS (Trimble Juno ST), using the free software CyberTracker (CyberTracker Software (Pty) Ltd Reg. no. 97/01908/07, http://www.cybertracker.co.za). Surveys were conducted daily during daylight hours (~ 12 hrs) at a mean vessel speed of 10 knots. During the surveys, two observers collected the data from both sides of the vessel, through naked eye scans supplemented by use of 7x50 binoculars with internal compass and reticle. A third observer assisted in the scans and recorded observations. Observers switched between the three positions at 2 hour intervals. Data recorded for each sighting included GPS position, date and time, sighting distance, sighting angle, species, group size and composition. Vessel speed, air temperature, wind speed and direction, Beaufort Sea State (BSS), cloud cover and visibility were recorded at the start of each day and updated whenever they changed.

Data were collected using line transect sampling methodology [38]. Both species appeared to be attracted to the ship (animals were frequently first seen on the vessel’s bow), violating the fundamental assumption of distance sampling, that animals are detected at their initial locations [38]. We therefore treated the data as if they were recorded from strip transects (assuming that all animals within the strip were detected [39]). Strip half-widths of 300 m for Commerson’s and 600 m for Peale’s dolphin were selected based on the frequency distribution of distances of sightings that could be recorded at their initial locations during this study (n = 23 and n = 79 for Commerson’s and Peale’s dolphin respectively). There were no sightings beyond 300m for Commerson’s dolphins and only two sightings beyond 600 m for Peale’s dolphins.

### Environmental data

The environmental variables were selected based on a combination of potential ecological significance and data availability. Bathymetry data were derived from ETOPO (ETopo Digital Maps) at a spatial resolution of 2’ (latitude and longitude). The nearest distance to coast was calculated using the Spatial Analyst tools in ArcGis 9.3 (ESRI). Monthly averages of sea surface temperature (SST) and of ocean colour (chlorophyll a, Chl*a*) were obtained from Aqua-MODIS (http://oceancolor.gsfc.nasa.gov), at 4km spatial resolution. Due to continuous cloud cover over the southern portion of study area, satellite data for these variables were not available at higher temporal resolutions. Marine Geospatial Ecological Tools for ArcGIS [40] was used to process the data and calculate the monthly averages of SST (°C) and Chl*a* (mg m^–3^).

### Data processing and analysis

Surveys used here were not specifically designed for recording cetaceans, because several research projects were simultaneously conducted onboard the research vessels.

#### Spatially explicit models

For analysis purposes, each day of survey effort was considered as a line transect. Each transect was divided into segments ~10 km long and 0.6 km or 1.2 km width for Commerson’s and Peale’s dolphins respectively. Spatial models of abundance for both species of dolphins were built using the ‘dsm’ package for R (http://github.com/DistanceDevelopment/dsm) [37]. Generalized additive models (GAMs) with negative binomial and Tweedie response distributions were fitted to investigate the relationships between the sightings and covariates for Commerson’s and Peale’s dolphin, respectively. Explanatory environmental variables were both static (geographic position, depth and distance to coast) and dynamic (SST and Chl*a*). Environmental variable values associated with each segment were obtained using ArcGis 9.3 (ESRI); the Lambert Azimuthal Equal-area (South Pole) projection was used. Bathymetry, Chl*a* and SST were calculated as mean values for the segment while distance to coast was measured to centroid of each segment. SST and Chl*a* values were estimated from contemporaneous satellite images (same year and month as each survey).

As the coast of the survey area includes peninsulas and gulfs, a soap film smoother [41] was used to model the complex coastline for the spatial term. All variables were fitted as smoothers. Interactions between explanatory variables were not evaluated in the models presented here.

Exploratory analysis was carried out to test correlation among explanatory variables [42]. The absolute values of correlation were always less than 0.35; therefore all variables were used in the global models. Models were constructed by a combination of forwards and backwards selection, removing non-significant smooth term (in function of approximate *p*-values, [43]) and adding one variable at each step.

Smoothness selection was performed by restricted maximum likelihood (REML) [44]. Models were checked using the ‘gam.check’ function and spatial autocorrelation in the residuals was evaluated using the ‘dsm.cor’ function, both in the ‘dsm’ package.

To predict abundance, grids of 3,143 and 6,611 predictive cells of 100 km^2^ were constructed using the ‘fishnet’ tool in ArcGis 9.3 for Commerson’s and Peale’s dolphin, respectively. For Peale’s dolphin, the prediction zone corresponded to the study area analyzed (Fig 1). For Commerson’s dolphin, however, the prediction zone was restricted to 200km from the shore, as most sightings of this species were coastal.

For dynamic environmental variables, a monthly average of data from November to April for each year (2009–2015) was used. Mean values of bathymetry, Chl*a* and SST were estimated for each grid cell and the centroid location of each cell was used to extract values from distance to coast and location (northing and easting).

The variance estimation of abundance models was obtained using methods detailed in [37, 45], which are included in the ‘dsm’ package. In order to estimate uncertainty per grid cell over multiple time periods, the usual rule that the variance of a sum is the sum of the covariances was used. For comparison we also calculated total abundances and maps of abundance and coefficient of variation per month.

## Results

**Fig 2:**
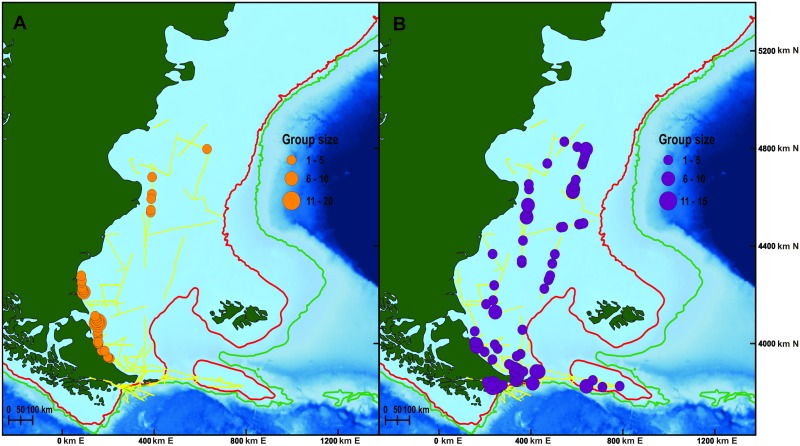
Dolphin sightings. Spatial distribution and size group of sightings of Commerson’s (A) and Peale’s (B) dolphin in relation to bathymetry from all scientific cruises surveys. Red line indicates the 200 m isobath. Green line indicates the 1000 m isobath.

Totals of 88 schools (212 individuals) of Commerson’s dolphin and 134 schools (465 individuals) of Peale’s dolphin were sighted in the 8,535 km surveyed over 55 days (Fig 2). Group size ranged from 1–20 (mean = 2; standard deviation, SD = 2.28; median = 2) for Commerson’s dolphin and from 1–15 (mean = 3.4, SD = 2.1, median = 3) for Peale’s dolphin. Most of the survey effort (85%) was conducted in shelf waters and all sightings of Commerson’s and 98.5% of sightings of Peale’s dolphin were recorded in waters < 200 m depth. Commerson’s dolphin was most frequently sighted close to shore (less than 60 km) while Peale’s dolphin occurred over a wider range of distances from the coast (~3.5 to 300 km; Fig 2). Temperatures where dolphins were present ranged from 7 to 14°C and 5 to 14.5°C for Commerson’s and Peale’s dolphin, respectively.

### Spatially explicit model and habitat preferences of Commerson’s dolphin

**Fig 3:**
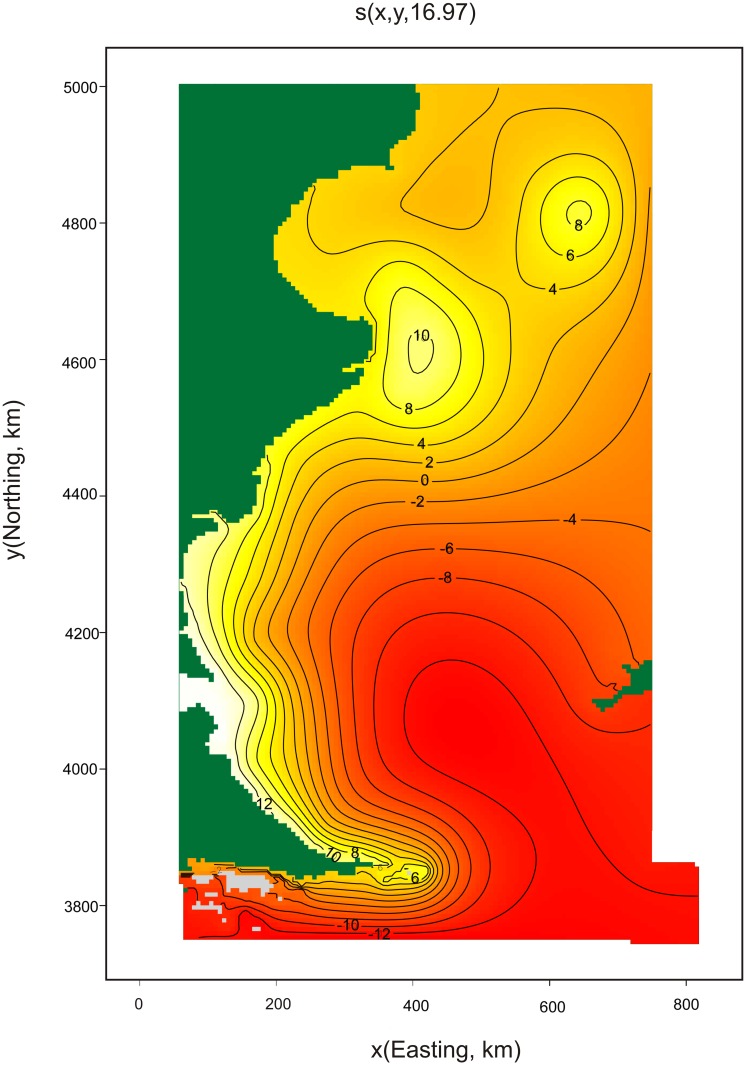
Relationships between the geographic position and the linear predictor in the model for Commerson’s dolphin. The number in brackets in “s” gives the effective degrees of freedom (a measure of flexibility) of each term. The contours (and colours) are the effect of the spatial smooth on abundance on the scale of the link function.

**Fig 4:**
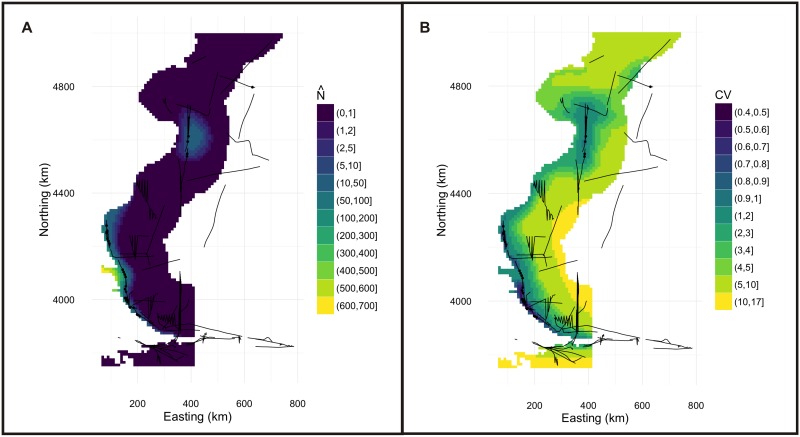
Spatially explicit model and uncertainty map of Commerson’s dolphin. Density surface model of abundance of Commerson’s dolphin in the total study area (a). This map indicate more dolphins near the coast, mainly in waters around Puerto Deseado (~ 4600 km northing, 400km easting) and South of Santa Cruz Province, Magellan Strait and San Sebastian Bay (~ 4100km northing, 100km easting). The map of per cell coefficient of variation (CV) for the fitted model shows a gradient of values increasing with increasing distance to coast (b). Cell area is 100km^2^.

**Fig 5:**
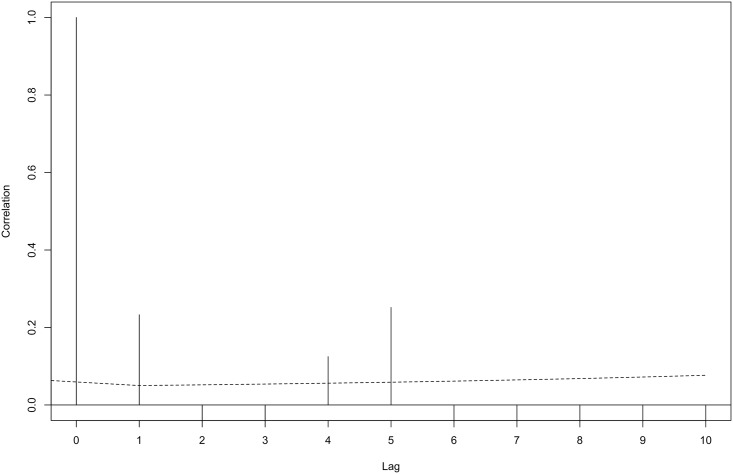
Autocorrelation of deviance residuals between segments (lags) for the fitted density surface model of Commerson’s dolphin. The dashed line represents the 95% confidence interval.

The final spatial model to predict Commerson’s dolphin abundance included only geographic position as explanatory variable, had an adjusted-R^2^ score of 0.159 and explained 83.4% of deviance. This model indicates that the abundance of Commerson’s is higher near the coast, mainly at 4600km northing and 4100km northing (Figs 2A, 3 and 4A). The estimated abundance was 21,933 individuals (% coefficient of variation (CV) = 74%, 95% confidence intervals (CI) = 6,013–80,012) in the studied region in summer-fall months. The model’s uncertainty (Fig 4B) revealed low per-cell CV values close to the coast and higher values in the rest of the area surveyed. Analysis showed little (˂0.3) spatial autocorrelation in the residuals (Fig 5).

### Spatially explicit model and habitat preferences of Peale’s dolphin

**Fig 6:**
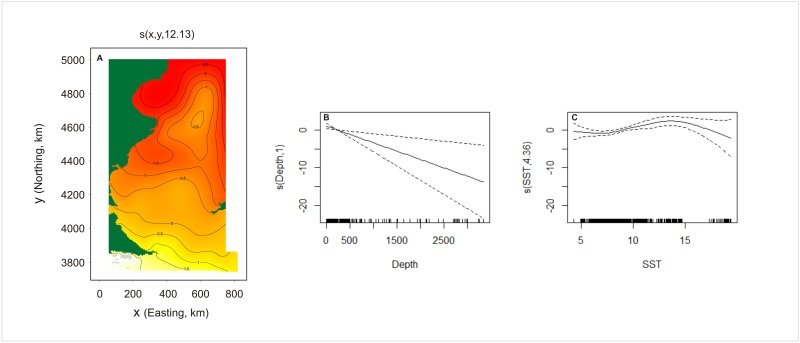
Relationships between the smooth terms and the linear predictor in the model for Peale’s dolphin. From left to right: (a) geographic position (easting, northing), (b) depth, and (c) sea surface temperature (SST). Dashed lines represent 95% confidence intervals. The number in brackets in each “s” gives the effective degrees of freedom (a measure of flexibility) of each term. The contours (and colours) are the effect of the spatial smooth on abundance on the scale of the link function.

**Fig 7:**
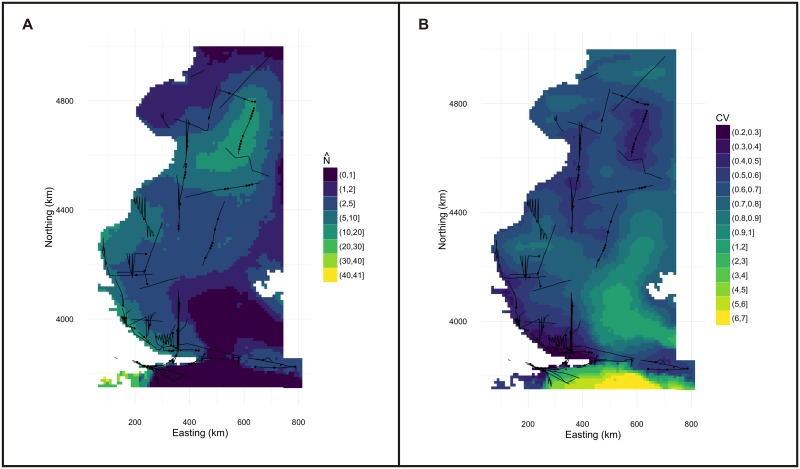
Spatially explicit model and uncertainty map of Peale’s dolphin. Density surface model of average abundance of Peale’s dolphin in the study area analyzed (a); averages were taken over the time periods where surveys were conducted, predicting using the appropriate dynamic variances for that time period. The map of per cell coefficient of variation (CV) for the fitted model shows the largest uncertainty in the southeast portion of the study area (b); uncertainty was combined over multiple time periods by noting that the variance of a sum is the sum of the covariances. Cell area is 100km^2^.

**Fig 8:**
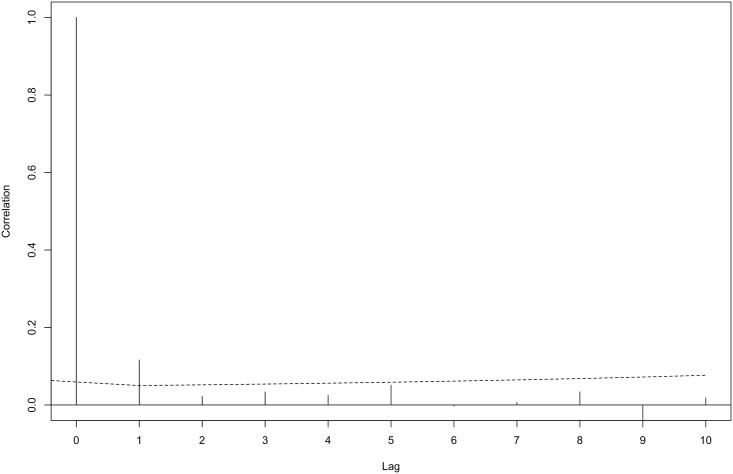
Autocorrelation of deviance residuals between segments (lags) for the fitted density surface model of Peale’s dolphin. The dashed line represents the 95% confidence interval.

The final spatial model for Peale’s dolphin abundance retained smooth terms for geographic position, depth and SST. Its adjusted-R^2^ score and percentage of deviance explained (0. 113 and 25.4% respectively) were lower than for the final model for Commerson’s dolphin. Peale’s dolphins were more numerous further south and in shallow waters with SST values between 9°C and 18°C (Fig 6). The fitted spatial model predicted an abundance of 19,924 individuals (CV = 19.6%, 95% CI = 12,878–30,823). Two hotspots are clearly distinguishable for Peale’s dolphin, one in central Patagonian waters, east of the San Jorge Gulf (~ 4600km northing to 4800km northing, 400km easting-600km easting) and the other one in the southern portion of the Tierra del Fuego Archipelago (~3700km northing-3800km northing; Fig 7A). Estimates with the highest uncertainty were found in the southern portion of the area covered in this study (Fig 7B). A small amount of unmodelled correlation in residuals was observed between adjacent segments in the fitted model (Fig 8).

Fig 7 summarizes the abundance and uncertainty over the time periods in which the surveys took place by averaging the abundance and computing the “average uncertainty” as detailed above. For comparison and to illustrate seasonal variations, S1, S2 and S3 Figs show the maps of abundance and coefficient of variation per month, as well as a time series (with confidence intervals) of the total abundance, respectively.

## Discussion

This study provides the first broad-scale abundance estimate for Peale’s dolphin in Argentine waters and an updated population size for Commerson’s dolphin in Patagonian Shelf waters. Using spatially explicit models, we identified hotspots of density and increased the knowledge of the ecological preferences of both species by surveying areas where similar studies had not been conducted before. The general spatial distribution of sightings as well as group sizes for both Commerson’s and Peale’s dolphins were consistent with previous studies on occurrence and distribution of both species conducted in areas where previous work overlapped this study [1, 6, 23, 46–48].

Peale’s dolphin had a wider offshore distribution than Commerson’s dolphin in all surveys where both were present. Similar results in other areas were found by White et al. [49], who reported a highly coastal distribution of Commerson’s dolphin during at-sea surveys around the Malvinas (Falkland) Islands, whereas Peale’s dolphin was found to have a more extensive distribution. Viddi et al. [27] also reported sightings of Peale’s dolphins close and far from shore in northern Patagonia, Chile.

Despite Chl*a* was not included as explanatory variable in the final model of both species, all sightings of both cetaceans were recorded in zones with median to high primary productivity. In addition, although relationships between abundance of these dolphins and frontal zones were not investigated here, the distribution of sighting of both species seems to match with frontal zones described in Acha et al. [50]. Commerson’s dolphin distribution overlaps with the Patagonian tidal frontal zone. An equivalent situation occurs with Peale’s dolphin records and the Patagonian cold estuarine zone. The occurrence of cetaceans in zones with high primary and secondary productivity has been widely documented in different regions of the world [35, 51–54].

Commerson’s and Peale’s dolphins were present mainly in shallow waters and their local abundance showed a negative relationship with depth (although this only was significant in the model for Peale’s dolphin), which is in accordance with studies by Goodall et al. [1, 6] and White et al. [49]. Nevertheless, these studies also reported sightings of Commerson’s [1] and Peale’s dolphins [6, 49] in areas which can only be reached by moving through deep waters (more than 200m), such as Namuncurá (Burdwood) Bank (~54° S to 54.5°S, 56°W to 62°W), Drake Passage and South Shetland Islands. For Commerson’s dolphin, most of these sightings should be considered as representing vagrants or at least outside of their normal distributional range since no new sightings of the species have been recorded further south of South America, despite numerous cruises around Antarctica ([1], NAD personal obs.).

Peale’s dolphins were found over the Namuncurá (Burdwood) Bank during November 2014 and February 2015 surveys. At present, with available data it is not possible to determine whether Peale’s dolphins remain in the Namuncurá (Burdwood) Bank area throughout the year or if animals move to other adjacent zones such as Malvinas (Falkland) Island and/or Tierra del Fuego. This question could be addressed with a greater number of systematic surveys of these areas in different seasons.

While the values of sea surface temperature in the study area ranged from ~ 4 to 19°C, dolphins were present at intermediate values. These ranges were similar to those reported in previous studies [1, 3, 6]. Since this study covers a wide latitudinal gradient, variation in SST values was often higher among transects within a survey than between months. SST was an important predictor of abundance for Peale’s dolphins and since they inhabit cold-temperate shelf waters, this relationship deserves special attention and predictive studies under different scenarios of environmental change involving increases in SST should be considered.

Abundance estimation obtained in this study could be biased given that dolphins (especially Commerson’s dolphins) showed a strong approaching behavior (hence no correction for incomplete detection was made), thus, our results should be considered with caution. In future studies, estimates positively biased as results of responsive movements towards the vessels could be corrected using the dual-platform approach suggested by Palka and Hammond [55].

Best and Halpin [56] suggest that spatial models could be less reliable than conventional distance sampling methods when there are many zeros in the data collected. This could be the case for the model fitted to the Commerson’s dolphin data, as uncertainty increased uniformly with increasing distance to coast, where no observations were recorded. The model for Peale’s dolphin revealed that uncertainty increased in unsampled deeper zones in the south-east portion of study area. Plotting the uncertainty in these areas is tricky as the coefficient of variation is the ratio of the standard deviation and abundance estimate. As the estimated abundance approaches zero, the CV can become very large (as we effectively divide by zero), so uncertainty in these areas (which can be located by looking at the abundance maps) should be interpreted with caution.

Despite the relatively poor precision of estimates from the spatial models (and that the model for Peale’s dolphin only explained 25% of deviance), the zones of higher densities identified for both species of dolphins were in agreement with the previous information for southwest South Atlantic Ocean such as Puerto Deseado and south of Santa Cruz Province, Magellan Strait and San Sebastian Bay for Commerson’s [1, 23, 24], and open waters east of San Jorge Gulf and southern Tierra del Fuego for Peale’s dolphin [2, 6], suggesting that this approach provides robust predictions of distribution.

The spatial modelling approach presented here is suited to the type of sighting data that can be obtained in multi-purpose research cruises, when survey designs for collecting sightings data are generally subordinate to other research tasks. This approach allows us to explore relationships between environmental covariates and the observed data. It is usually expected that a model-based approach to abundance estimation leads to smaller uncertainty estimates than design-based estimates [37]. Even when this is not the case, the spatial nature of the abundance estimates and the benefits from presentation as maps are extremely useful.

Abundance estimates provided in this study were based on surveys conducted during summer-fall months. Similar studies in winter months should be considered since seasonal changes in distribution and abundance have previously been suggested for both dolphin’ species [1, 3, 6, 13, 23, 27, 47–49]. Inshore/offshore movements following prey have been proposed for Commerson’s dolphin in the southern part of its distribution, with the species moving into deeper waters during cold months [1, 9, 19, 20]. Stable isotope analysis of dolphin bones suggested that, in Tierra del Fuego, Commerson’s dolphin consumes pelagic and benthopelagic prey in coastal and continental shelf waters [57]. Stable isotopes in bones integrate several (5–10) years of feeding information [58] and thus dolphins could be using coastal and continental waters either in different years or within a year in different seasons. In addition, in northern and central Patagonia, seasonal movements were related to temperature variations [48] and reproductive behavior [23] respectively.

Winter offshore movements were also suggested for Peale’s dolphin in Tierra del Fuego, Argentina [6] although seasonal movements are better documented for Chilean populations. During land-based surveys along the west side of the Strait of Magellan, Lescrauwaet [47] found an increase in abundance during summer whereas the number of animals was greater during spring in the northern Patagonian fjords [27].

This study covered a wide range of the distribution of both dolphins in the Patagonian shelf, representing the first large-scale study on the spatial distribution, overall abundance estimates and habitat preferences for Peale’s dolphin in the southwest South Atlantic Ocean and the first of this kind at the southern boundary of the distribution of Commerson’s dolphins.

Large-scale studies are important not only to better understand the variables affecting the distribution and abundance of species but also to determine the environmental conditions which define the edges of their ranges. This knowledge is particularly important to predict the potential ecological responses of species to threats such as global climate change or habitat degradation [15, 32]. Considering that Commerson’s and Peale’s dolphins are listed as “Data Deficient” by the IUCN [16, 17] and that, given their coastal habits, they are exposed to numerous threats, this type of information can be useful for wildlife managers to help design conservation strategies such as identifying priority areas for conservation.

## Supporting Information

S1 Fig Map of estimated abundance for each month when surveys were conducted for Peale's dolphin. Each estimate was calculated using dynamic covariate values averaged over the month in question. Distribution (though not necessarily magnitude) is relatively consistent over time. Survey lines and observations are overlaid.
(TIF)

S2 Fig Map of coefficient of variation for each month when surveys were conducted for Peale's dolphin. Each estimate was calculated using dynamic covariate values averaged over the month in question. Survey lines and observations are overlaid. Uncertainty is highest in unsurveyed areas and lowest where survey effort was expended, in the month it was expended.
(TIF)

S3 Fig Estimates of abundance (points) and corresponding confidence intervals (lines) for each month when surveys were conducted for Peale's dolphin. Each estimate was calculated using dynamic covariate values averaged over the month in question.
(TIF)
