# Supplementary figures and images for: Spatial Models of Abundance and Habitat Preferences of Commerson’s and Peale’s Dolphin in Southern Patagonian Waters
Source: PLoS One. 2016 Oct 26;11(10):e0163441. doi: 10.1371/journal.pone.0163441 (PMC5082685; doi:10.1371/journal.pone.0163441)

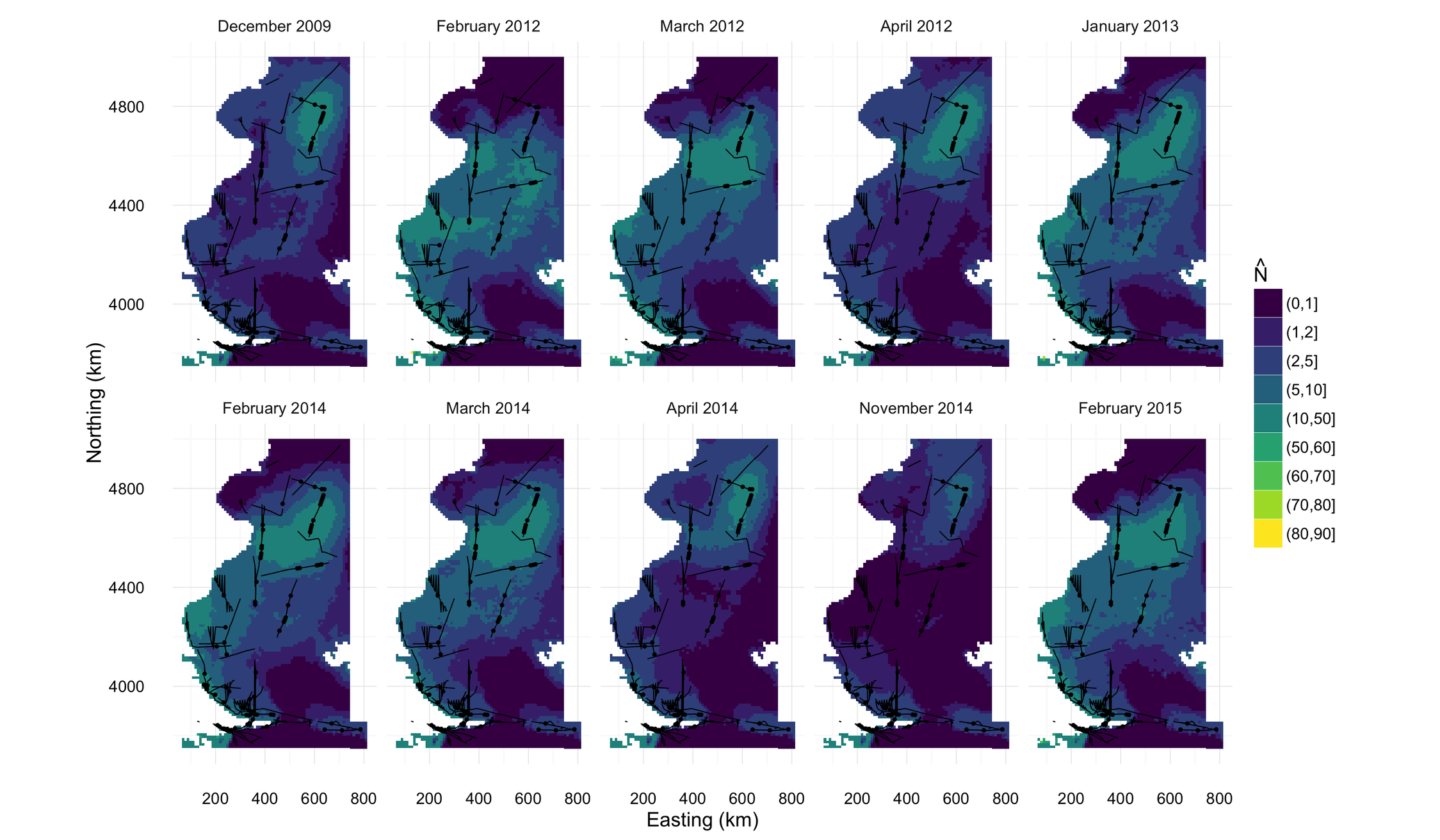

Supplement: S1 Fig — Each estimate was calculated using dynamic covariate values averaged over the month in question. Distribution (though not necessarily magnitude) is relatively consistent over time. Survey lines and observations are overlaid. (TIF) [file pone.0163441.s001.tif]

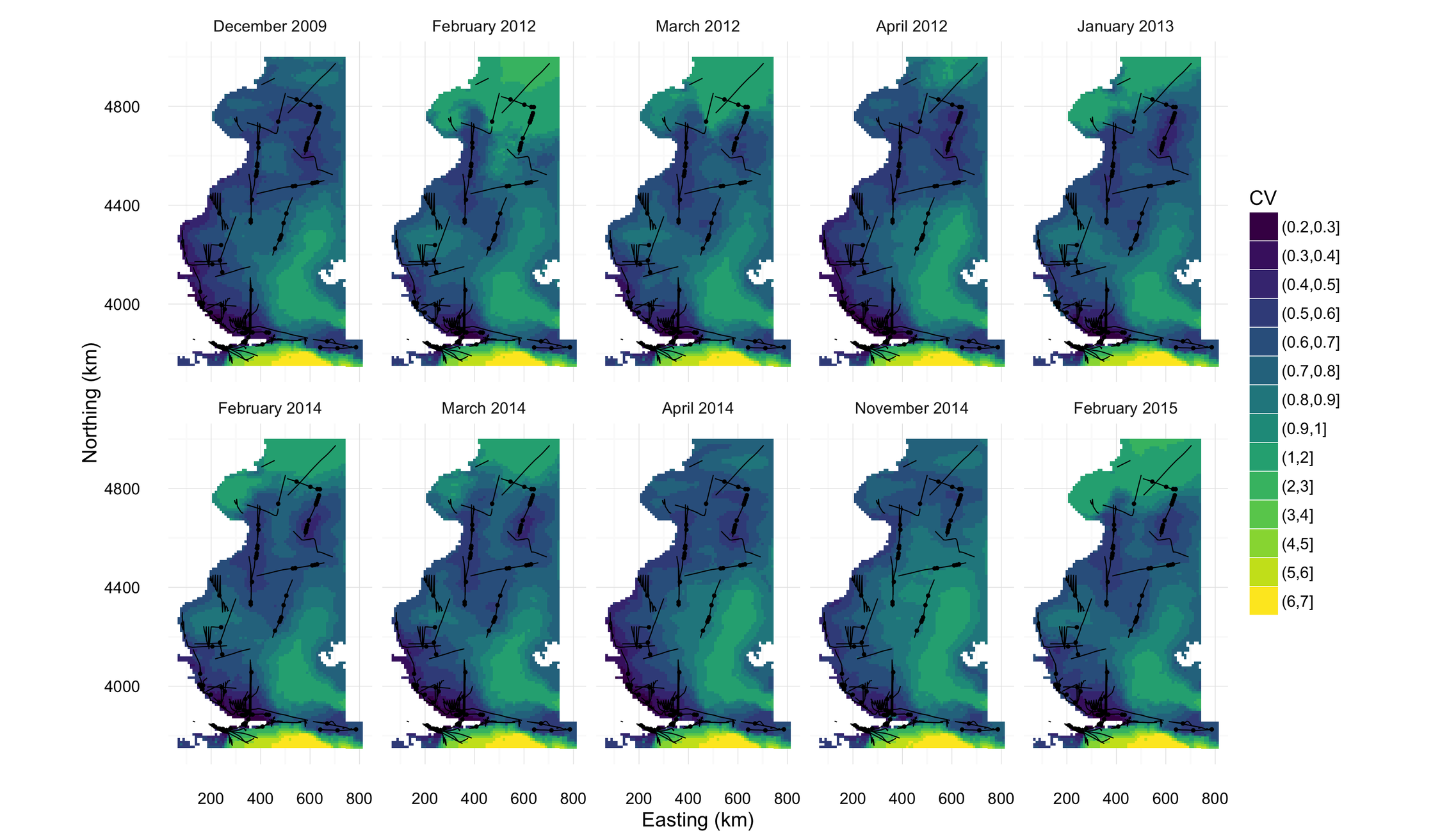

Supplement: S2 Fig — Each estimate was calculated using dynamic covariate values averaged over the month in question. Survey lines and observations are overlaid. Uncertainty is highest in unsurveyed areas and lowest where survey effort was expended, in the month it was expended. (TIF) [file pone.0163441.s002.tif]

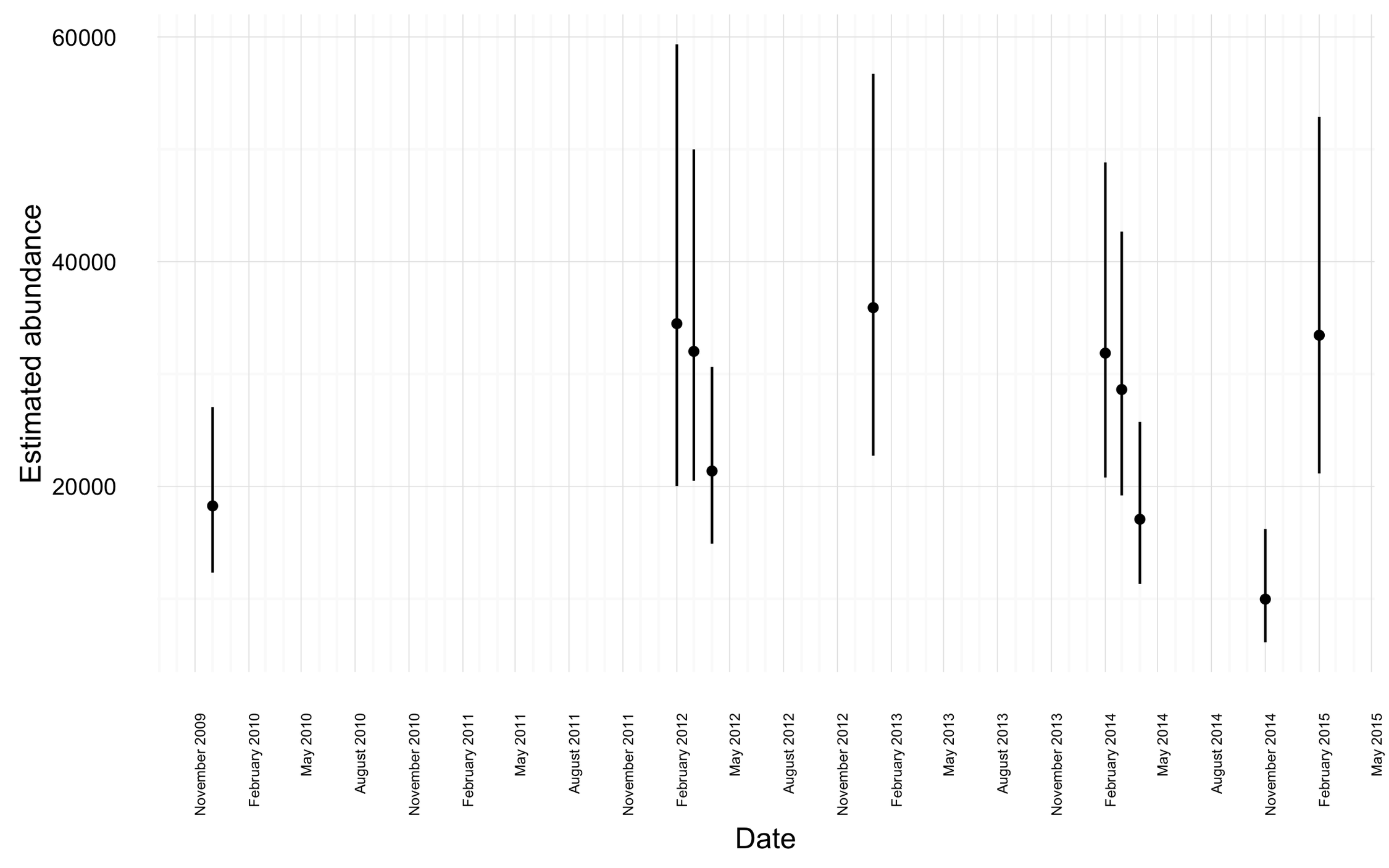

Supplement: S3 Fig — Each estimate was calculated using dynamic covariate values averaged over the month in question. (TIF) [file pone.0163441.s003.tif]
